# Netrin-1 Peptide Is a Chemorepellent in* Tetrahymena thermophila*

**DOI:** 10.1155/2016/7142868

**Published:** 2016-03-31

**Authors:** Heather Kuruvilla, Bradley Schmidt, Stephanie Song, Marian Bhajjan, Matthew Merical, Caleb Alley, Christopher Griffin, David Yoder, Josephine Hein, Daniel Kohl, Cambria Puffenberger, David Petroff, Elise Newcomer, Kortney Good, Graham Heston, Anna Hurtubise

**Affiliations:** Cedarville University, Cedarville, OH 45314, USA

## Abstract

Netrin-1 is a highly conserved, pleiotropic signaling molecule that can serve as a neuronal chemorepellent during vertebrate development. In vertebrates, chemorepellent signaling is mediated through the tyrosine kinase, src-1, and the tyrosine phosphatase, shp-2.* Tetrahymena thermophila* has been used as a model system for chemorepellent signaling because its avoidance response is easily characterized under a light microscope. Our experiments showed that netrin-1 peptide is a chemorepellent in* T. thermophila* at micromolar concentrations.* T. thermophila *adapts to netrin-1 over a time course of about 10 minutes. Netrin-adapted cells still avoid GTP, PACAP-38, and nociceptin, suggesting that netrin does not use the same signaling machinery as any of these other repellents. Avoidance of netrin-1 peptide was effectively eliminated by the addition of the tyrosine kinase inhibitor, genistein, to the assay buffer; however, immunostaining using an anti-phosphotyrosine antibody showed similar fluorescence levels in control and netrin-1 exposed cells, suggesting that tyrosine phosphorylation is not required for signaling to occur. In addition, ELISA indicates that a netrin-like peptide is present in both whole cell extract and secreted protein obtained from* Tetrahymena thermophila.* Further study will be required in order to fully elucidate the signaling mechanism of netrin-1 peptide in this organism.

## 1. Introduction

The netrin family of proteins are highly conserved pleiotropic signaling molecules which belong to the laminin superfamily [1]. Netrins are present in all bilaterally symmetrical animals studied to date, and receptors for netrins have been found in all vertebrates studied thus far [2]. The most well-characterized netrin, netrin-1, binds to four different classes of receptors: deleted in colorectal cancer (DCC), uncoordinated 5 homolog (UNC-5), Down's syndrome cell adhesion molecule (DSCAM), and neogenin [1–3]. Netrin-1 was first noted for its effects on axonal guidance [4, 5]. When signaling through DCC, DSCAM, or neogenin, netrin-1 may act as a chemoattractant; however, when signaling through UNC-5 or a heterodimer of DCC and UNC-5, netrin acts as a chemorepellent [1–3]. In addition to playing a chemotactic role, netrin-1 and its receptors have been implicated in angiogenesis, apoptosis, and tumor suppression [1–3, 6].

Much effort has gone into understanding the various signaling pathways through which netrin-1 works. Signaling mechanisms are complex, given the number of receptors netrin-1 binds to, as well as the multifaceted physiological roles attributed to netrin-1. Chemoattractant signaling is thought to involve a large number of growth and survival pathways, along with focal adhesion kinase, while chemorepellent signaling is thought to involve the tyrosine kinase, src-1, and the tyrosine phosphatase, shp-2 [1, 2]. At present, chemorepellent signaling is less well understood than chemoattractant signaling.

*Tetrahymena thermophila* is free-living, eukaryotic ciliates which have been used as a model system for chemorepellent signaling for several decades [7–11]. When exposed to a chemorepellent,* Tetrahymena* reverse their cilia, causing them to exhibit avoidance behavior which is characterized by swimming in circles or jerky, back and forth swimming. This behavior is easily observed and characterized under a light microscope.* Tetrahymena* avoid a number of polycationic proteins and peptides that are derived from vertebrate systems, including lysozyme [9], PACAP-38 [12], and nociceptin [13]. This avoidance may sometimes be blocked using pharmacological inhibitors, allowing us to determine which signaling pathways are necessary for avoidance [12, 13].

Since netrin-1 acts as a chemorepellent in some vertebrate cell types, and since netrin-1 peptide is polycationic under our assay conditions, we believe that netrin-1 peptide will act as a chemorepellent in* Tetrahymena thermophila.*

## 2. Materials and Methods

### 2.1. Cell Cultures

*Tetrahymena thermophila*, strain B2086.2, was obtained from the Tetrahymena Stock Center at Cornell University https://tetrahymena.vet.cornell.edu/. Cells were grown at 20°C in the medium of Dentler [15] without shaking or addition of antibiotics. One-day-old cultures were used for all behavioral and pharmacological assays.

### 2.2. Chemicals and Solutions

Netrin-1 peptide and anti-netrin-1 antibody were obtained from Abcore LLC, Ramona, CA. PACAP-38 was obtained from the American Peptide Company, Sunnyvale, CA. GTP-*γ*-S and pharmacological inhibitors were obtained from Tocris Bioscience, Minneapolis, MN, USA. Anti-*α*-tubulin antibody was obtained from Abcam, Cambridge, MA, USA. QuantaRed*™* enhanced chemifluorescent HRP substrate was obtained from Pierce Biotechnology, Rockford, IL, USA. All other chemicals were obtained from Sigma Chemical Co., St. Louis, MO, USA.

Phosphate buffered saline (PBS) was made by dilution of a 10x stock with distilled water. Behavioral buffer consisted of 10 mM Trizma Base, 0.5 mM MOPS, and 50 *μ*M CaCl_2_; pH was adjusted to 7.0 with HCl. Blocking buffer consisted of 3% bovine serum albumin (BSA) and 0.3% Triton X-100, diluted in 1x PBS. Antibody dilution buffer consisted of 1% bovine serum albumin (BSA) and 0.3% Triton X-100, diluted in 1x PBS.

### 2.3. Behavioral Assays

Behavioral assays were carried out as previously described [8, 9, 11–13].* Tetrahymena thermophila* was washed 3 times in behavioral buffer and then 300 *μ*L of cell suspension was transferred to the first well of a microtiter plate. Cells were then transferred individually using a micropipette into the second well of the microtiter plate, already containing 300 *μ*L of buffer as a control. Cells were then transferred to a third well containing 300 *μ*L of netrin-1 peptide. Behavior of the cells was observed (1–5 seconds), and the percentage of cells exhibiting avoidance behavior was noted. Varying concentrations of each peptide were used until we determined the minimum concentration at which 100% of the cells exhibited avoidance behavior (EC_100_). In order to minimize variability due to cells conditioning the buffer, cells were used within 30 minutes after being washed in buffer.

Adaptation assays were carried out as previously described [9]. Cells were placed in 1 *μ*M netrin-1 peptide for a specified amount of time. Cells were then washed in buffer for 20 seconds and then transferred back into 1 *μ*M netrin-1 peptide to assay for repellent response. Cells were then individually scored for avoidance.

Cross-adaptation assays were carried out as previously described [16]. Cells were first placed in one of the test solutions for 10 minutes, until the cells were adapted to that solution. They were then washed briefly (20 seconds) in buffer and transferred to another test solution. Cells were then individually scored for avoidance.

Pharmacological inhibition assays were performed similar to the behavioral assays described above. After being washed in buffer, cells were exposed to pharmacological agents known to block specific signaling pathways and incubated for 15 minutes. Cells were then transferred to a solution containing netrin-1 peptide at EC_100_ and then monitored for avoidance behavior.

### 2.4. Immunofluorescence

*T. thermophila* were washed 3 times in behavioral buffer and then fixed in 3.7% formaldehyde, diluted in behavioral buffer, for 15 minutes. Cells were then washed 3 times in PBS and incubated in blocking buffer at room temperature, overnight with shaking. Cells were then washed 3 times in PBS and incubated in a 1 : 100 dilution of either anti-netrin-1 or anti-tubulin antibody in the presence of antibody dilution buffer, for 2 hours at room temperature, with constant shaking. Cells were once again washed 3 times in PBS and then incubated with a 1 : 100 dilution of secondary antibody, in the presence of antibody dilution buffer for one hour at room temperature, with constant shaking. Cells were then washed three times in PBS, stained with DAPI, and viewed under a Nikon H550L Microscope using the Nikon Intensilight C-HGFI. Fluorescence images were obtained with a QI Click 74-0083-AO camera using NIS Elements BR 4.13.04 Software.

### 2.5. ELISA

One-day-old cultures of* T. thermophila* were washed in behavioral buffer consisting of 1 mM Tris, 0.5 mM MOPS, and 50 mM CaCl_2_; pH 7.0. 10 mL of culture was concentrated into 1 mL of buffer. For secreted proteins, cells were incubated at room temperature for 24 hours with constant shaking. The cells were then pelleted by centrifugation and the supernatant was saved as our secreted protein sample. For the whole cell extract, cells were incubated on ice in 1% Triton X-100 for one hour in the presence of protease inhibitor cocktail. Both extracts were used in ELISA, using 1 : 10,000 dilution of goat anti-netrin-1 IgG as the primary antibody and a 1 : 5,000 dilution of rabbit-anti-goat IgG, HRP conjugate, as the secondary antibody. QuantaRed*™* enhanced chemifluorescent HRP substrate was used to develop the ELISA, and the plate was read using a Promega Glomax Multi Detection System.

## 3. Results and Discussion

Netrin-1 is a basic peptide, with a net charge of +6 at pH 7.0. The amino acid sequence of this peptide is shown below:

 
**K**FQQ**R** *E* **KK**G**K**C**KK**A.

**Figure 1:**
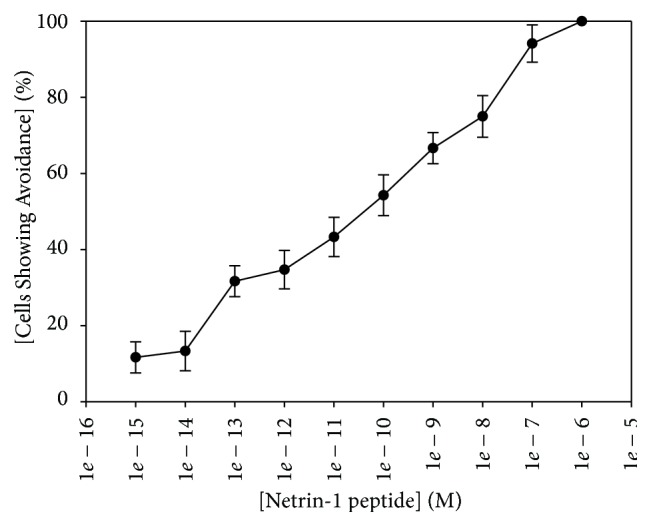
Netrin-1 peptide is a chemorepellent in* Tetrahymena thermophila.* The EC_100_ of this peptide is 1 *μ*M. The EC_50_ of this peptide is approximately 1 nM. “Cells Showing Avoidance” represents the mean of at least 6 trials, and error bars represent the standard deviation. Each trial consisted of 10 cells which were individually observed and scored for avoidance within the first 5 seconds of exposure to netrin-1 peptide.

Basic amino acids are shown in bold, while acidic amino acids are shown in italic. The net charge of the peptide is +6 at our assay pH of 7.0. In our behavioral assay, netrin-1 peptide was a chemorepellent (Figure 1), consistent with our hypothesis and with netrin-1 activity in other cell types [4, 17]. Cells showed avoidance over a broad range of concentrations, with an EC_100_ of 1 *μ*M, and an EC_50_ of approximately 1 nM (Figure 1). In contrast, similar concentrations of myelin basic protein (4–14), which has a charge of +4 under our assay conditions, did not elicit avoidance behavior (data not shown). The EC_100_ of netrin-1 peptide is lower than what we have previously described for both lysozyme and nociceptin, which have an EC_100_ of approximately 100 *μ*M (Table 3), but higher than what we have observed for PACAP-38 (0.1 *μ*M; Table 3). We were able to obtain a small quantity of recombinant human netrin-1 and found that* Tetrahymena thermophila* avoided this compound as well (data not shown). However, since netrin-1 peptide was more readily obtained and elicited robust avoidance, we chose to use netrin-1 peptide for the remainder of our experiments.

**Figure 2:**
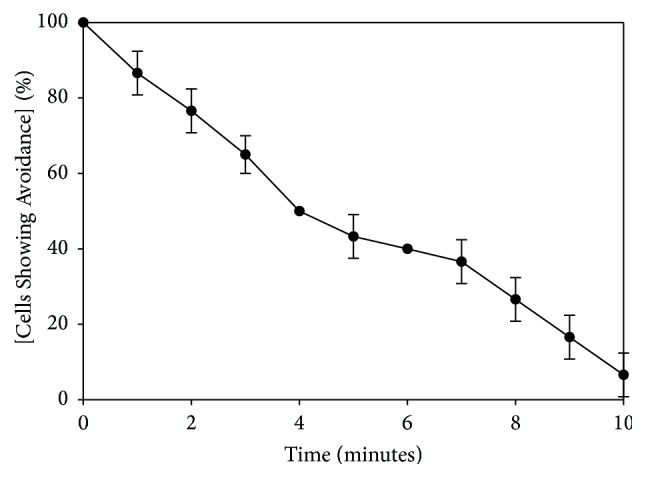
Time course of adaptation to netrin-1 peptide in* Tetrahymena thermophila.* Adaptation studies were done at 1 *μ*M netrin-1 peptide, which is the EC_100_ of this peptide. “Cells Showing Avoidance” represents the mean of at least 6 trials, and error bars represent the standard deviation. Each trial consisted of 10 cells which were individually observed and scored for avoidance.

*Tetrahymena* adapt to netrin-1 peptide over the course of about 10 minutes (Figure 2), which is similar to the time course of adaptation to the chemorepellents lysozyme [9], GTP [9], and PACAP-38 [16]. Cross-adaptation assays (Table 1) show that netrin-1 adapted cells still avoid PACAP-38, nociceptin, and GTP. Similarly, cells that adapted to any of these other compounds still show avoidance to netrin-1 (Table 1), suggesting that netrin-1 signaling is using a different receptor and/or second messenger pathways than those used by the other three repellents. Controls (netrin-1 adapted to netrin-1, PACAP adapted to PACAP, and GTP adapted to GTP) showed only baseline avoidance, indicating that adaptation to these compounds was occurring. Baseline avoidance in our behavioral studies indicates the amount of avoidance typically seen when moving cells from a well containing behavioral buffer to another well containing the same buffer and ranges from about 7 to 20% ([8, 11, 12], current study). This is how baseline avoidance is defined in the pharmacological studies as well.

**Figure 3:**
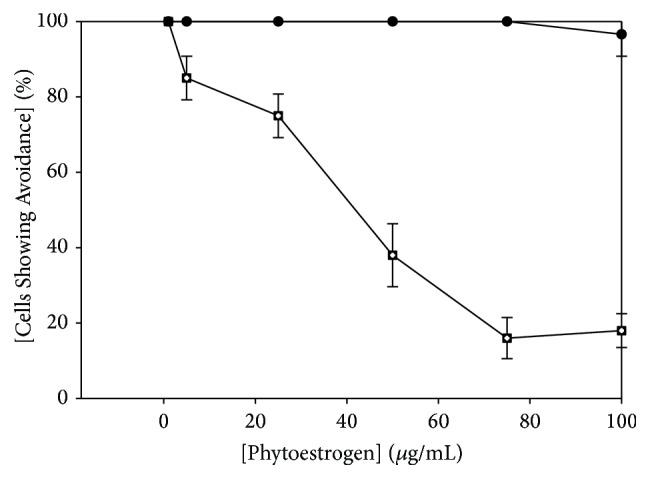
Avoidance of netrin-1 peptide is inhibited by the tyrosine kinase inhibitor, genistein (closed squares), but not by daidzein (closed circles). The IC_50_ of genistein is approximately 50 *μ*g/mL. Baseline avoidance was achieved at a genistein concentration of 75 *μ*g/mL. Daidzein had no effect on avoidance behavior. Percentages represent the mean ± standard deviation of at least 6 trials. Each trial consisted of 10 cells which were individually observed and scored for avoidance.

In order to determine the signaling mechanism of netrin-1 peptide in* Tetrahymena thermophila*, we used a broad spectrum of pharmacological inhibitors targeting a number of second messenger pathways that have previously been associated with netrin signaling in vertebrates. Since netrin-1 mediated repellent signaling in vertebrates involves src-1 and shp-2 [1, 2], we included tyrosine kinase inhibitors and tyrosine phosphatase inhibitors in our study (Table 2). We also included a number of inhibitors targeting G-protein linked receptors and associated pathways, as well as focal adhesion kinases, since these have been associated with netrin-1 signaling in some systems [1, 2, 18]. As seen in Table 2, none of these compounds effectively reduced netrin-1 avoidance, with the exception of the tyrosine kinase inhibitor, genistein. The phytoestrogen, genistein, inhibited avoidance to netrin-1 peptide at concentrations ranging from 20 to 100 *μ*g/*μ*L (Table 2; Figure 3). Avoidance was reduced to baseline levels at 75 *μ*g/*μ*L genistein; the IC_50_ of genistein was approximately 50 *μ*g/*μ*L (Figure 3). These IC_50_ values are similar to those we have previously seen when inhibiting GTP avoidance [11]. Another phytoestrogen, daidzein, was used as a negative control for genistein. Daidzein did not affect netrin-1 avoidance (Figure 3), indicating that genistein's effects were specific to that compound.

**Figure 4:**
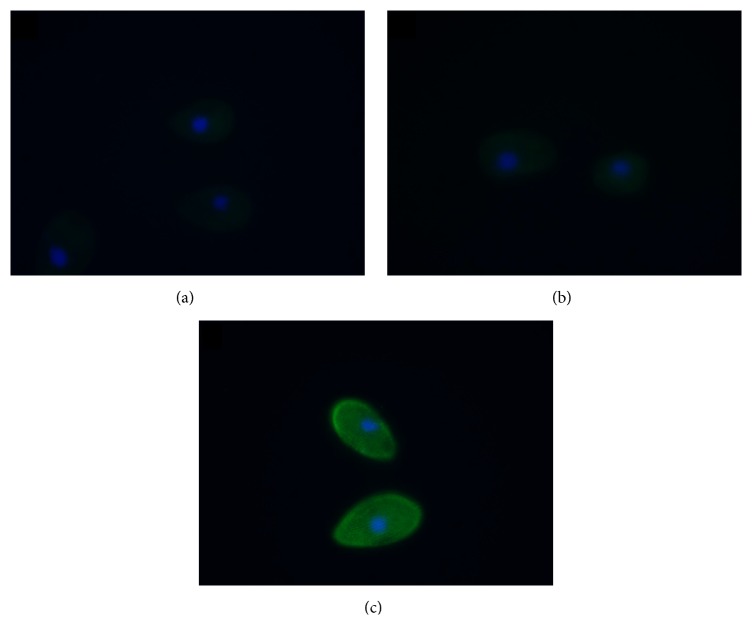
Tyrosine phosphorylation levels are not affected by netrin-1 peptide. Indirect immunofluorescence using PT-66 anti-phosphotyrosine antibody shows no difference in fluorescence intensity between control (a) and netrin-1 exposed cells (b). This indicates that tyrosine phosphorylation is not required for netrin-1 signaling. In contrast, cells stained with an anti-tubulin antibody (c) show a high level of fluorescence intensity.

Immunolocalization of phosphotyrosine in control (a) and netrin-1 peptide (b) treated cells showed low levels of phosphotyrosine staining in both treatment groups (Figure 4). The anti-tubulin control (c) showed much higher levels of staining than either of the phosphotyrosine treatment groups. Treatment with netrin-1 peptide did not increase phosphotyrosine levels in* Tetrahymena*, suggesting that tyrosine phosphorylation was not required for netrin-1 peptide signaling. This is consistent with the findings of Eisen et al. [19]. When Eisen and colleagues sequenced the* Tetrahymena* genome, they found no consensus sequences for tyrosine kinases [19]. However, other studies [11, 20] have implicated tyrosine kinase signaling in* Tetrahymena*, both on the basis of pharmacological inhibition by genistein and on the basis of immunolocalization of phosphotyrosine. While our immunofluorescence data does not show an increased level of phosphorylation in the presence of netrin-1 peptide, genistein does inhibit netrin-1 avoidance. Together, these data suggest that genistein may have a different mechanism of action in* Tetrahymena* than it does in vertebrates; however, our experiments do not reveal what this mechanism might be. It is possible that genistein is simply blocking another kinase; however, the broad spectrum kinase inhibitors used in our assay had no effect on the avoidance of netrin-1 peptide (Table 2). It is also possible that genistein is binding to another enzyme or second messenger involved in netrin-1 signaling. Additional experiments will be required in order to determine the mechanism of genistein action in this organism.

Since calcium-based depolarizations have been implicated in* Tetrahymena* avoidance [13, 14, 21], and since calcium has been implicated in netrin signaling [22], we used chelators of intracellular and extracellular calcium to try to inhibit avoidance. EGTA was used to chelate extracellular calcium, and thapsigargin was used to deplete calcium from intracellular stores. Neither chelator affected avoidance to netrin-1 peptide (Table 2). Table 3 compares three polycationic compounds that are chemorepellents in* Tetrahymena.* Calcium is required for the avoidance of all of the compounds listed except for netrin-1 peptide. Calcium-based depolarizations have previously been shown in the presence of lysozyme [14] and nociceptin [13] as well as the repellents GTP [10] and ATP [21]. The absence of calcium dependence in the avoidance of netrin-1 peptide suggests a calcium-independent signaling mechanism for ciliary reversal that has not previously been described in this organism, which is unlikely to involve phosphorylation (Table 2).

Netrin-1 peptide has one of the lowest EC_100_ values of the polycationic compounds studied in* Tetrahymena thermophila* to date (Table 3), suggesting a relatively high affinity for its putative receptor when compared with other chemorepellents in this organism. However, pharmacological studies lead us to believe that the* Tetrahymena* receptor is unlikely to show conservation with the previously described vertebrate receptors, since blocking signaling pathways used in netrin-1 signaling in vertebrates did not impact the avoidance of netrin-1 peptide in* Tetrahymena* (Table 2).

The physiological role of netrin-1 peptide in* Tetrahymena thermophila* is also an open question.* Tetrahymena thermophila* is thought to respond to chemorepellents in an attempt to escape predation and possibly to warn other cells of danger [7, 13]. We hypothesized that* Tetrahymena* might secrete a netrin-1 like protein in order to communicate with other cells. Our ELISA data (Table 4) indicate that a netrin-like protein is present in both whole cell extracts and secreted protein obtained from* Tetrahymena thermophila*. The secreted protein also caused avoidance behavior in* Tetrahymena thermophila *(data not shown). Since netrin-like protein appears to be biologically active in terms of causing avoidance, it is likely that this protein is used in intercellular communication. We are currently doing experiments to further characterize this netrin-1-like protein. Though netrin appears to be conserved throughout the animal kingdom [2], this is the first time such a protein has been described in kingdom Protista.

Further studies are needed in order to elucidate the signaling mechanism of netrin-1 peptide in* Tetrahymena thermophila*, as well as to characterize the netrin-like protein that is secreted by* Tetrahymena*. Additional data will help us understand whether* Tetrahymena* could be a useful model system for netrin-1 signaling in vertebrates. In addition, since most chemorepellents in* Tetrahymena* require calcium in order for avoidance to occur, understanding netrin-1 signaling may uncover a previously unknown mechanism for ciliary reversal in* Tetrahymena thermophila.*

## Figures and Tables

**Table 1:** Cells that adapted to netrin-1 peptide are not cross-adapted to nociceptin, GTP, or PACAP-38. Percentage of avoidance, as listed below, represents the mean ± standard deviation of at least 6 trials. Each trial consisted of 10 cells which were individually observed and scored for avoidance within the first 5 seconds of exposure to the chemorepellent being tested. Adaptation to the same signal (e.g., GTP adapted to GTP, nociceptin adapted to nociceptin) was run as controls. Each of these controls showed adaptation, showing less than the baseline avoidance of 20% typically seen in behavioral assays.

	GTP	PACAP-38	Nociceptin	Netrin-1 peptide
GTP	13.3 ± 5.8	95.0 ± 5.0	94.0 ± 5.2	93.0 ± 4.1
PACAP	96.6 ± 5.2	12.5 ± 9.6	97.5 ± 4.6	95.0 ± 5.5
Nociceptin	96.6 ± 5.8	100 ± 0.0	9.2 ± 8.2	95.0 ± 5.0
Netrin-1 peptide	90.0 ± 10.0	90.0 ± 0.0	93.3 ± 5.8	6.66 ± 5.8

**Table 2:** Pharmacological studies suggest that a tyrosine kinase is involved in the avoidance of netrin-1 peptide in *Tetrahymena*. Inhibition of G-proteins, chelation of intracellular and extracellular calcium, and inhibition of a number of other kinases had no effect on avoidance behavior. The tyrosine kinase inhibitor, genistein, reduced avoidance to baseline levels. “Avoidance” represents the mean ± standard deviation of 6 trials. Each trial consisted of 10 cells which were individually observed and scored for avoidance within the first 5 seconds of exposure to netrin-1 peptide.

Pharmacological agent	Mechanism of action	Concentration tested	Avoidance
EGTA	Chelates extracellular calcium	100 *μ*M	100 ± 0%
BAPTA-AM	Chelates intracellular calcium	100 *μ*M	96.6 ± 5.8%
Thapsigargin	Depletes ER calcium stores	100 *μ*M	100 ± 0%
GDP-*β*-S	Inhibits G-proteins	1 mM	93.3 ± 5.8%
Pertussis toxin	Inhibits Gi/o proteins	0.2 *μ*g/mL	96.6 ± 5.8%
Rp-cAMPs	Inhibits protein kinase A	100 *μ*M	93.3 ± 5.8%
U73122	Inhibits phospholipase C	1 *μ*M	95.0 ± 5.0%
Genistein	Inhibits tyrosine kinases	100 *μ*g/mL	16.7 ± 4.5%
Daidzein	Negative control for genistein	100 *μ*g/mL	98.3 ± 4.1%
Neomycin sulfate	Competitive inhibitor for lysozyme/PACAP receptor	100 *μ*M	93.3 ± 5.8%
SU6668	Protein kinase Inhibitor	100 *μ*M	100 ± 0%
NS2028	Guanylyl cyclase inhibitor	100 *μ*M	100 ± 0%
GSK429286	Rho kinase inhibitor	100 *μ*M	100 ± 0%
Src inhibitor 1	Inhibits Src family kinases	100 *μ*M	100 ± 0%
FAK inhibitor 14	Inhibits focal adhesion kinase	100 *μ*M	93.3 ± 5.8%
Sodium orthovanadate	Inhibits tyrosine phosphatases	100 *μ*M	97.5 ± 5.0%

**Table 3:** A comparison of polycationic peptides and proteins which are chemorepellents in *Tetrahymena*. Calcium is required for avoidance of all of the compounds listed except for netrin-1 peptide, suggesting a unique signaling mechanism for this peptide. Netrin-1 peptide also has one of the lowest EC_100_ values of the polycationic compounds studied to date, suggesting a high affinity for its putative receptor.

Chemorepellent	Charge at pH 7.0	EC_100_	Calcium required for avoidance?	Reference
Lysozyme	+11	100 *μ*M	Yes^*∗*^	Kuruvilla et al., 1997 [9]; Kuruvilla and Hennessey, 1998 [14]
PACAP-38	+11	0.1 *μ*M	Yes^+^	Mace et al., 2000 [12]
Nociceptin	+4	100 *μ*M	Yes^*∗*^	Lampert et al., 2013 [13]
Netrin-1 peptide	+6	1 *μ*M	No^+^	Current study

^*∗*^Electrophysiological studies done. ^+^Results obtained using pharmacological inhibitors only.

**Table 4:** ELISA indicates that a netrin-like protein is secreted by *Tetrahymena thermophila*. A polyclonal anti-netrin-1 antibody showed reactivity in ELISA with whole cell extract as well as secreted protein obtained from *Tetrahymena thermophila*. Using a standard curve of netrin-1 concentration, we found that both total protein and secreted protein had a netrin-like peptide concentration of approximately 0.1 *μ*M.

Protein extract	Total protein concentration	Concentration of netrin-like peptide
Whole cell extract	114 mg/mL	0.1 *μ*M
Secreted protein	0.29 mg/mL	0.1 *μ*M

## References

[1] Ko S. Y., Dass C. R., Nurgali K. (2012). Netrin-1 in the developing enteric nervous system and colorectal cancer. *Trends in Molecular Medicine*.

[2] Sun K. L. W., Correia J. P., Kennedy T. E. (2011). Netrins: versatile extracellular cues with diverse functions. *Development*.

[3] Yung A. R., Nishitani A. M., Goodrich L. V. (2015). Phenotypic analysis of mice completely lacking Netrin-1. *Development*.

[4] Colamarino S. A., Tessier-Lavigne M. (1995). The axonal chemoattractant netrin-1 is also a chemorepellent for trochlear motor axons. *Cell*.

[5] Ming G.-L., Song H.-J., Berninger B., Holt C. E., Tessier-Lavigne M., Poo M.-M. (1997). cAMP-dependent growth cone guidance by netrin-1. *Neuron*.

[6] Castets M., Mehlen P. (2010). Netrin-1 role in angiogenesis: to be or not to be a pro-angiogenic factor?. *Cell Cycle*.

[7] Rodgers L. F., Markle K. L., Hennessey T. M. (2008). Responses of the ciliates Tetrahymena and Paramecium to vertebrate odorants and tastants. *Journal of Eukaryotic Microbiology*.

[8] Robinette E. D., Gulley K. T., Cassity K. J. (2008). A comparison of the polycation receptors of *Paramecium tetraurelia* and *Tetrahymena thermophila*. *Journal of Eukaryotic Microbiology*.

[9] Kuruvilla H. G., Kim M. Y., Hennessey T. M. (1997). Chemosensory adaptation to lysozyme and GTP involves independently regulated receptors in *Tetrahymena thermophila*. *Journal of Eukaryotic Microbiology*.

[10] Kim M. Y., Kuruvilla H. G., Hennessey T. M. (1997). Chemosensory adaptation in paramecium involves changes in both repellent binding and the consequent receptor potentials. *Comparative Biochemistry and Physiology Part A: Physiology*.

[11] Bartholomew J., Reichart J., Mundy R. (2008). GTP avoidance in *Tetrahymena thermophila* requires tyrosine kinase activity, intracellular calcium, NOS, and guanylyl cyclase. *Purinergic Signalling*.

[12] Mace S. R., Dean J. G., Murphy J. R., Rhodes J. L., Kuruvilla H. G. (2000). PACAP-38 is a chemorepellent and an agonist for the lysozyme receptor in *Tetrahymena thermophila*. *Journal of Comparative Physiology A*.

[13] Lampert T., Nugent C., Weston J., Braun N., Kuruvilla H. (2013). Nociceptin signaling involves a calcium-based depolarization in *Tetrahymena thermophila*. *International Journal of Peptides*.

[14] Kuruvilla H. G., Hennessey T. M. (1998). Purification and characterization of a novel chemorepellent receptor from *Tetrahymena thermophila*. *Journal of Membrane Biology*.

[15] Dentler W. L. (1988). Fractionation of Tetrahymena ciliary membranes with Triton X-114 and the identification of a ciliary membrane ATPase. *The Journal of Cell Biology*.

[16] Keedy M., Yorgey N., Hilty J., Price A., Hassenzahl D., Kuruvilla H. (2003). Pharmacological evidence suggests that the lysozyme/PACAP receptor of *Tetrahymena thermophila* is a polycation receptor. *Acta Protozoologica*.

[17] Eichmann A., Noble F., Autiero M., Carmeliet P. (2005). Guidance of vascular and neural network formation. *Current Opinion in Neurobiology*.

[18] Liu G., Beggs H., Jürgensen C. (2004). Netrin requires focal adhesion kinase and Src family kinases for axon outgrowth and attraction. *Nature Neuroscience*.

[19] Eisen J. A., Coyne R. S., Wu M. (2006). Macronuclear genome sequence of the ciliate *Tetrahymena thermophila*, a model eukaryote. *PLoS Biology*.

[20] Christensen S. T., Guerra C. F., Awan A., Wheatley D. N., Satir P. (2003). Insulin receptor-like proteins in *Tetrahymena thermophila* ciliary membranes. *Current Biology*.

[21] Kim M. Y., Kuruvilla H. G., Raghu S., Hennessey T. M. (1999). ATP reception and chemosensory adaptation in *Tetrahymena thermophila*. *The Journal of Experimental Biology*.

[22] Hong K., Nishiyama M., Henley J., Tessler-Lavigne M., Poo M.-M. (2000). Calcium signalling in the guidance of nerve growth by netrin-1. *Nature*.

